# Omics Sciences in Dentistry: A Narrative Review on Diagnostic and Therapeutic Applications for Prevalent Oral Diseases

**DOI:** 10.3390/diagnostics15233086

**Published:** 2025-12-04

**Authors:** Marco Lollobrigida, Giulia Mazzucchi, Alberto De Biase

**Affiliations:** Department of Oral and Maxillofacial Sciences, Sapienza University of Rome, 00161 Rome, Italy

**Keywords:** omics sciences, omics, genomics, transcriptomics, proteomics, metabolomics, dentistry

## Abstract

Omics sciences are revolutionizing the field of biomedical and dental research by allowing for an integrated understanding of the molecular basis of health and disease. This narrative review analyzes the role of these novel technologies supporting the diagnosis, prognosis, and treatment of the most noteworthy oral diseases, such as dental caries, periodontitis, and oral squamous cell carcinoma. The review discusses the characterization of disease-associated genetic variations and polygenic risk scores as evidenced by genomic studies. It further examines how transcriptomic analyses can identify diagnostic gene expression signatures of immune dysregulation and tumor heterogeneity. The contribution of proteomics and metabolomics studies to the discovery of diagnostic and prognostic protein and metabolites biomarkers is also analyzed. Finally, the integration of different omics datasets within multi-omics frameworks is discussed as a key approach for a holistic interpretation of oral pathogenesis and data-driven precision dentistry. The review also addresses current limitations in the clinical translation of omics sciences into tools for early diagnosis, personalized prevention, and targeted therapy.

## 1. Introduction

The emergence of the omics sciences, traditionally represented by genomics, transcriptomics, proteomics, and metabolomics, is progressively transforming biomedical research by for allowing an unprecedented understanding of the molecular basis of both health and disease. Omics sciences are based on technologies that, in one experiment, can analyze the genome, transcriptome, proteome, and metabolome, essentially spanning the gap between genotype and phenotype.

In dentistry, prevalent conditions such as dental caries and periodontitis arise from complex and dynamic interactions among genetic predisposition, microbial communities, environmental influences, and lifestyle factors. Oral squamous cell carcinoma (OSCC) and other mucosal diseases also depend on complex etiopathogenic mechanisms involving genetic factors, environmental exposures, metabolic alterations, and immune system interactions. Within this multifactorial context, omics sciences provide powerful tools to unravel intricate molecular networks, enabling the identification of novel diagnostic biomarkers and potential therapeutic targets. Beyond their individual contributions, the integration of omics sciences within multi-omics frameworks also offers a holistic perspective on oral disease biology, facilitating precision diagnostics and personalized therapeutic strategies.

This narrative review aims to provide an overview of the existing evidence on the application of omics sciences to dental practice. Each of the omics disciplines will be examined, with an assessment of key findings related to major oral diseases, while also considering prospective directions for research and clinical application. We will discuss their separate and combined roles in advancing a data-driven, patient-centered approach to dentistry. While the oral microbiome is a key player in several oral diseases, its comprehensive discussion falls beyond the primary scope of this review, whose focus is on the host-centric omics signatures.

A literature review was conducted by searching an electronic database (MEDLINE, PubMed) for relevant primary studies and review articles published in peer-reviewed journals over the past decade. The search strategy employed the following key terms as text words: (“omics” OR “omics sciences” OR “genomics” OR “transcriptomics” OR “proteomics” OR “metabolomics”) AND (“oral” OR “caries” OR “periodontitis” OR “autoimmune”, OR “OSCC”). In addition to the database search, citation searching was performed on the reference lists of all included studies to identify additional relevant articles.

## 2. Genomics in Oral Health and Disease

**Figure 1:**
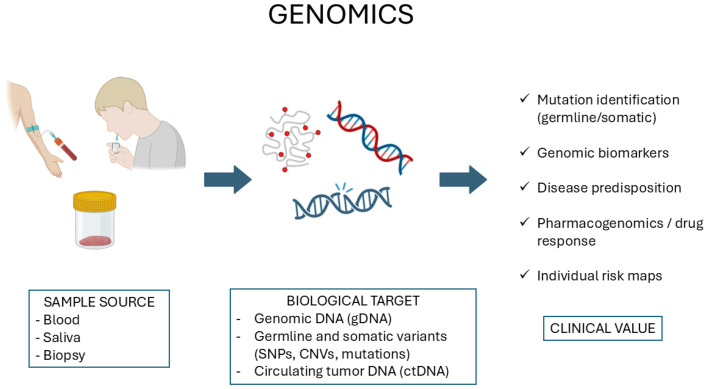
Genomic analyses applied to dentistry and oral medicine assess DNA from blood, saliva, or tissue to identify genetic variants. The resulting data provides insights into disease susceptibility, somatic mutations, and potential genomic biomarkers.

Genomics involves the thorough analysis of an entire organism’s complete DNA, consisting of coding as well as non-coding sequences [1]. Genomics has advanced significantly following the completion of the Human Genome Project [2], while new technologies like next-generation sequencing (NGS), whole-exome sequencing (WES), and single-nucleotide polymorphism (SNP) genotyping have become more common. These technologies essentially assess mutations, structural variation, as well as gene–environment interactions responsible for disease etiology (Figure 1).

In dentistry, genomics highlights the genetic variants that are responsible for susceptibility to oral diseases. For instance, Genome-Wide Association Studies (GWAS) can provide specific polygenic risk scores (PRS) [3] associated with increased susceptibility to conditions like periodontitis and dental caries, helping to explain why, even with similar hygiene habits, some people are at a much higher risk. On the other hand, functional genomics is actively exploring the gene expression and regulation to decipher the underlying biological mechanisms of oral tissue homeostasis and disease [4].

### 2.1. Genomics of OSCC

OSCC is the most common cancer of the oral cavity, characterized by aggressiveness, local recurrence tendencies, and high mortality. Despite refinements of surgery, radiotherapy treatments, and chemotherapy regimens, the five-year survival rate of OSCC has changed very little over the past few decades [5], mostly because of advanced stages at presentation. In this context, genomics has emerged as an effective tool for defining the molecular landscape of OSCC, offering hope for early diagnosis, predictive testing of recurrence/progression risk, and of personalized treatment strategies.

Comprehensive genome profiling analyses have revealed a mutational complexity in OSCC. Mutations have been recurrently identified within the genes TP53, CDKN2A, NOTCH1, FAT1, and CASP8 [6,7], each of them involved with tumor suppressor activity, cell cycle control, and apoptosis. Of these, TP53, encoding the tumor suppressor protein p53, is the most mutated gene encountered within OSCC cases; mutations here are frequently found within high-grade tumors with poor prognosis. CDKN2A, encoding the p16INK4a protein, is frequently silenced by promoter hypermethylation or mutations that result in aberrantly progressing cells. Alterations of NOTCH1, a gene involved with cell differentiation and stem cells’ maintenance, highlight the context-dependent duality of genetic mutations in tumorigenesis (tumor promoter versus tumor suppressor activity) depending on cellular environment. Similarly, mutations in FAT1 and CASP8 underscore the environment-dependent duality of genetic alterations in OSCC. FAT1 mutations often disrupt cell adhesion and polarity, promoting oncogenic signaling, while CASP8 alterations can impair apoptotic responses or, conversely, drive pro-inflammatory and pro-survival pathways within the tumor microenvironment. Although TP53, CDKN2A, NOTCH1, FAT1 and CASP8 are consistently identified as key genes involved in the pathogenesis of oral squamous cell carcinoma, their precise biological and clinical roles, particularly their prognostic value, remain partly controversial, due to differences in genetic alteration, population background, tumor subsite and methodological settings.

The RAS family of proto-oncogenes (including HRAS, KRAS, and NRAS) plays a significant, though less frequent, role in the pathogenesis of OSCC [8]. These genes encode GTP-binding proteins that are critical regulators of cell proliferation, survival, and differentiation. In OSCC, activating point mutations in RAS genes, most notably in HRAS, lock the protein in an active GTP-bound state, leading to persistent signaling through downstream pathways like the MAPK/ERK cascade, independent of external growth signals. The therapeutic utility of directly targeting RAS in OSCC, however, is limited because RAS mutations are relatively rare and, when present, most frequently involve H-RAS and non-G12C variants, rendering current KRAS G12C-specific inhibitors largely ineffective [9].

Beyond these well-characterized drivers, sequencing efforts have identified recurrent mutations in other tumor suppressor genes, including protein tyrosine phosphatase, receptor type T (PTPRT) [10], which regulates STAT3 signaling and cell growth, and AT-rich interaction domain 2 (ARID2) [11], a component of the SWI/SNF chromatin remodeling complex, highlighting the importance of dysregulated signaling and epigenetic control in OSCC pathogenesis.

Population-specific genome variations add complexity to the OSCC mutational profile. For instance, mutations at the TERT promoter that enhance telomerase activity and impart replicative immortality are higher in selected Asian populations [12]. This geographical variation highlights how environmental exposures may interact with the cellular genome, potentially through epigenetic mechanisms, to shape distinct paths of cancer development.

Beyond somatic mutations, infection with high-risk Human Papillomavirus (HPV), particularly HPV-16, represents a fundamental etiological factor for a distinct subclass of head and neck carcinomas [13]. HPV-positive tumors possess an extraordinarily stable genome with a low mutational burden, as the viral oncoproteins E6 and E7 drive tumorigenesis by inactivating the key cellular tumor suppressors p53 and pRB, respectively [14]. This alternative carcinogenic pathway results in a significantly more favorable clinical and prognostic profile compared to HPV-negative tumors driven by gene mutations and exposure to smoke and alcohol. The p16^INK4a^ protein, encoded by the CDKN2A gene, serves as a clinically established and robust surrogate biomarker for oncogenic HPV infection [15]. The overexpression of p16, detectable by immunohistochemistry on tumor tissue, is a direct consequence of the functional loss of pRB. P16 immunohistochemistry has then become a routine diagnostic and prognostic test, enabling the stratification of OSCC patients into distinct clinical cohorts. This highlights how genomic alterations, even of viral origin, are directly translatable into diagnostic tools that guide patient management.

Third-generation sequencing platforms allow to subclassify OSCC into molecular subcategories with separate biology and therapeutically targetable vulnerabilities [16]. For example, tumors that have PI3KCA mutations or PTEN loss have activation of the PI3K/AKT/mTOR pathway that provides a basis for pathway-specific inhibitors of targeted therapy [17]. In addition, mutations of genes involved in the immune checkpoint control like CD274 (PD-L1) amplification or JAK1/2 mutations have provided opportunities for immunotherapeutic approaches like immune checkpoint inhibitors [18].

A major frontier in current research is the development of liquid biopsy techniques. These approaches are expected to integrate genomic data with clinical practice by allowing for the non-invasive identification of tumor-exclusive mutations in circulating tumor DNA (ctDNA) [19]. The potential applications of this technology include monitoring minimally residual disease, detecting recurrence, and assessing response to therapy at population-level. In addition, PRS and germline variant analysis could one day enable early identification of individuals at high risk of OSCC, thus guiding enhanced surveillance and early interventions [20].

Despite such progress, the translation of genome findings into routine clinical application is still limited. While biomarkers like HPV status have been successfully translated, the use of broader mutational signatures or PRS is not yet standardized. Tumor heterogeneity at both inter-tumoral as well as intra-tumoral levels could affect the predictive potential of single gene mutations. Epigenetic modulation, non-coding RNAs, and gene–environment interactions need also to be completely elucidated. Big data-dependent multi-ethnic genome-wide analyses and systems biology approaches are necessary for elucidating the of OSCC pathogenesis and building predictive models. For these reasons, the routine clinical application of comprehensive genomic profiling for diagnosis, therapy selection, and prognosis in OSCC remains limited.

### 2.2. Genomics of Dental Caries

Dental caries is a complex, multifactorial disease resulting from the interaction of microbial activity, host properties, dietary habits, and external factors. Current genomic explorations are increasingly demonstrating that genetic susceptibility of hosts can affect personal responses to cariogenic stimuli.

Over the past decade, GWAS have identified numerous genetic loci for caries susceptibility of primary and permanent teeth. One of the most remarkable findings has been the gene WNT10A. This gene encodes a protein active in the WNT/β-catenin signaling pathway that is crucial for odontogenesis processes as well as enamel mineralization [21]. Gene mutants have been related to enamel hypoplasia and then enhanced caries susceptibility due to the compromised structure of the enamel [22]. However, the presence of enamel hypoplasia due to gene mutation does not automatically equate to high caries risk while its clinical significance is highly dependent on the extent and severity of the defect.

In addition to WNT10A, other loci have been found to be of significance [3,23,24], including genes involved in immune regulation (e.g., DEFB1), salivary chemistry (e.g., AQP5), and carbohydrate metabolism (e.g., TAS2R38). These findings highlight the polygenic nature of caries susceptibility, indicating that multiple low-effect genetic loci contribute to disease risk alongside environmental factors such as dietary sugar intake and fluoride availability.

Notably, genetic variations in taste receptors, particularly the sweet taste receptor genes TAS1R2 and TAS1R3, provide a direct molecular link between genotype and dietary exposure [25]. Certain alleles of these genes, in fact, were found to be associated with altered sweet taste perception and preference. Individuals with a genetically determined higher preference for sweetness may unconsciously consume more sugary foods and beverages, thereby significantly increasing their caries risk. This illustrates a compelling pathway where host genetics indirectly influences disease susceptibility by shaping behavioral patterns, a crucial consideration for personalized dietary counseling and preventive strategies.

Beyond innate genetic variation, evidence also points to the role of early-life nutritional programming in shaping sweet taste preference and, subsequently, caries risk. According to this concept, exposure to high levels of sugars during critical developmental windows in infancy and childhood can alter the perception of and preference for sweet tastes [26]. Emerging research suggests that these long-lasting effects are mediated, at least in part, by epigenetic mechanisms. A diet high in sugars during early life can induce epigenetic modifications in genes involved in taste transduction pathways (e.g., TAS1R2, TAS1R3) and in the central reward circuits of the brain [27,28]. These epigenetic changes are believed to “recalibrate” the hedonic and perceptual response to sweetness, leading to a heightened preference for intensely sweet foods and a reduced satisfaction from less sweet options. In general, epigenetic control could explain differences in caries experience between individuals who share the same genetic background but who have been exposed to different environment factors [29].

Functional genomics analyses have provided more insight by looking at the effects of genetic variants on gene expression and protein activity of oral tissues. As a case in point, mutations of the AMELX gene that encodes for amelogenin have been linked with dysregulated enamel matrix production and increased lesion development [30]. Similarly, mutations that play a role in matrix metalloproteinase expression (e.g., MMP20) have been linked with dysregulated enamel maturation and carious lesion progression [31].

An interesting study on twins by Gomez et al. [32] has demonstrated the influence of genetics on microbiome composition. The authors observed that host genetics can dictate the composition of the supragingival plaque microbiome but, notably, the “heritable” bacterial taxa were not those associated with dental caries, which, on the other hand, were primarily affected by environmental factors. This convergent approach has the potential of overcoming predictive models of caries susceptibility based only on genetic or only on microbiological factors.

In clinical practice, the development of PRS from identified risk alleles opens the door for personalized prevention of caries. In a large-scale study by Fires et al. [33], the predictive value of a PRS for caries was examined across over 15,000 Swedish adults. The authors found a statistically significant association between high PRS and an increased caries burden, as measured by decayed, missing, and filled surfaces (DMFS). This pioneering work represents a critical first step, paving the way for validation in larger and more diverse cohorts.

In conclusion, it is crucial to recognize that the genetic associations described above are not deterministic. The presence of a risk allele signifies a probabilistic increase in susceptibility, but the ultimate phenotypic expression, namely the development of caries, is modulated by environmental and behavioral factors (diet, fluoride and oral hygiene). In many individuals, a favorable environment can effectively compensate for a high genetic risk, while a cariogenic diet can cause disease even in a genetically resistant background. Consequently, common preventive strategies, including dietary counseling and fluoride-based prevention, are universally beneficial, cost-effective, and recommended for all patients, irrespective of their genetic profile.

### 2.3. Genomics of Periodontal Disease

Periodontitis is a chronic inflammatory disease resulting in the progressive loss of the supporting structures of the tooth including the periodontal ligament and the alveolar bone. While traditionally considered as the result of bacterial infection, there is mounting evidence that genetic factors play a crucial role in disease susceptibility, progression and response to treatment. Although the heritability of periodontitis appears lower than that of other multifactorial diseases, genetic predisposition was found to contribute to inter-individual variation in disease outcomes and response to treatment [34].

GWAS have identified multiple loci that may play a role in susceptibility to periodontitis. Of particular consistency is the association with variants of the SIGLEC5 gene. The gene encoding the sialic acid-binding immunoglobulin-like lectin is expressed upon immune cells as an inhibitory receptor that balances inflammatory reactions [35]. The polymorphism rs12461706 of SIGLEC5 has been linked with increased risk of periodontitis, presumably by interfering with resolution of inflammation in the periodontal environment.

Other genetic factors contributing to susceptibility to periodontitis include those influencing innate immune mechanisms and inflammatory processes, such as variants in interleukin (IL)-1, IL-6, TNF-α, and TLR4. Polymorphisms of the IL1 gene cluster, most significantly of IL-1α and IL-1β, have been related to enhanced interleukin production and increased clinical attachment loss [36]. Other genetic polymorphisms involved could be those in the vitamin D receptor gene (VDR) and the matrix metalloproteinase genes MMP-2, MMP-8, MMP-9, which are implicated in bone resorption processes and extracellular matrix remodeling processes [37,38]. However, these findings remain difficult to interpret with confidence, as many reported associations show limited reproducibility across studies and populations.

An emerging area of research highlights the possible connection between metabolic hormone pathways and periodontal inflammation. Given the well-established bidirectional relationship between periodontitis and diabetes mellitus [39], genetic variations in genes encoding receptors for metabolic hormones have been investigated for their role in periodontal susceptibility. Notably, polymorphisms in the gene encoding the Glucagon-Like Peptide-1 Receptor (GLP-1R) have been implicated [40]. GLP-1 is an incretin hormone that stimulates insulin secretion and possesses potent anti-inflammatory properties. Genetic variants in GLP-1R that reduce signaling efficacy may not only predispose individuals to type 2 diabetes but also impair the local anti-inflammatory response in periodontal tissues facilitating exaggerated inflammation and alveolar bone loss. While this hypothesis offers an appealing explanation for the clinical comorbidity observed between metabolic and periodontal diseases, current evidence remains preliminary and requires research.

Functional genomics approaches have suggested that polymorphisms associated with periodontitis susceptibility may influence gene expression patterns, particularly within immune cell populations. In this context, several risk alleles are linked to enhanced inflammatory gene expression or increased transcriptional responses to microbial stimuli, thereby potentially increasing an individual’s tendency toward chronic periodontal inflammation [41]. However, the prevailing model of immune hyper-responsiveness as the primary driver of pathology is contrasted by evidence suggesting that immunodeficiency and impaired bacterial clearance can similarly lead to destructive inflammation, highlighting the complexity and heterogeneity of the disease. In addition, epigenetic changes like DNA methylation and histone alterations are also increasingly recognized as crucial regulators of the occurrence of periodontal disease. Environmental factors like tobacco use, poor oral hygiene, and systemic conditions like diabetes mellitus can induce epigenetic modifications that regulate the expression of immunity-related genes and alter disease susceptibility without changing the underlying DNA sequence [42].

In recent years, integrated studies combining genomics with oral microbiome analyses have begun to better elucidate host–microbiota interactions. Host genetic variation may influence the subgingival biofilm composition and pathogenicity by changing the expression of antimicrobial peptides and immune receptors [43]. This interaction highlights the need for system-level approaches that integrate both microbial and host dynamics.

Even with such advancements, numerous challenges remain. Genetic heterogeneity of periodontitis and environmental factors pose challenges for both the interpretation and reproducibility of genetic studies. Furthermore, most of the studies have been carried out on populations of European descents, highlighting the need for more comprehensive investigations of the genetic variance existing across populations globally.

From a clinical standpoint, genomics has the potential to define the risk of individuals as well as personalized treatments of periodontal diseases, particularly for patients who do not respond to conventional therapy. Nevertheless, translating genomics discoveries into clinical periodontology still requires validation and, not least, assessment of cost-effectiveness. Presently, the primary value of periodontitis genomics remains the elucidation of pathogenic mechanisms, providing a molecular framework for understanding the disease’s inter-individual variability.

### 2.4. Summary and Future Directions

Genomic studies have established a hereditary component in the susceptibility and progression of major oral diseases (Table 1). For OSCC, this translates into defined mutational landscapes and viral etiologies (HPV) that are already informing or may inform diagnostic, therapeutic and prognostic strategies. In dental caries and periodontitis, GWAS have revealed a polygenic basis of risk, implicating genes involved in enamel formation, taste perception, and immune response. The future of genomic dentistry will be integrating these findings into risk scores to identify high-risk individuals as well as to deliver personalized therapies. However, overcoming challenges related to population diversity and clinical cost-effectiveness remains a crucial step.

## 3. Transcriptomics and Gene Expression Profiling

**Figure 2:**
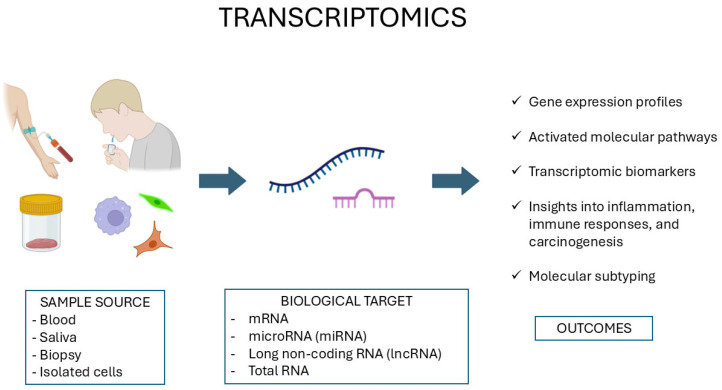
Transcriptomic profiling of blood samples, saliva and tissues reveals gene expression changes involved in oral inflammation, carcinogenesis, host–microbe interactions, and tissue remodeling. These data may provide molecular insights and candidate biomarkers for the diagnosis and stratification of oral diseases.

Transcriptomics involves an integrated analysis of the complete set of RNA transcripts, the transcriptome, produced by the genome under defined physiological or disease conditions [44]. The transcriptome consists of a diverse array of RNA species, including messenger RNAs (mRNAs) that encode proteins, in addition to non-coding RNAs such as microRNAs (miRNAs) [45] and long non-coding RNAs (lncRNAs) [46], which are involved at multiple levels of gene expression regulation. Unlike the relatively stable genome, the transcriptome is dynamic and externally responsive to external stimuli, thus providing an essential biomarker of cellular status, differentiation processes, and overall functional status (Figure 2).

Numerous strategies have been conceived to characterize the transcriptome. Early approaches, such as microarray technologies, enabled the simultaneous detection of thousands of preselected transcripts. These methods are increasingly being replaced by RNA sequencing (RNA-seq), which offers an overall and unbiased description of the transcriptome that identifies unknown transcripts as well as variants of alternative splicing [47]. More recently, single-cell RNA sequencing (scRNA-seq) has revolutionized the field by enabling gene expression profiling at single-cell resolution [48], revealing cellular heterogeneity and rare subpopulations that were previously masked in bulk tissue analyses.

In oral disease research, expression signatures characteristic of conditions such as autoimmune mucosal disease and OSCC and have been discovered [49,50,51,52,53]. Additionally, temporal transcriptomic studies aim to track changes in gene expression over the course of disease progression or in response to treatment [54], providing kinetic insights into biological processes and drug efficacy. On this basis, transcriptomics holds a direct translational potential, greater than that of genomics, since it captures the active molecular drivers of disease in real-time. By defining the specific inflammatory pathways (e.g., IFN-α, IL-17, TNF-α) and cellular states (e.g., T-cell exhaustion) active in each patient, transcriptomic profiling can directly indicate targeted biological therapies.

### 3.1. Transcriptomics of Autoimmune Oral Diseases

Autoimmune oral diseases are a group of chronic inflammatory diseases characterized by a maladaptive immune response of the host that results in destruction of the oral mucosal lining. Well-studied examples include oral lichen planus (OLP), pemphigus vulgaris (PV), mucous membrane pemphigoid (MMPg) and Sjögren’s syndrome (SS). For all these diseases, transcriptomics has provided detailed insights into the immune mechanisms, cellular heterogeneity, and molecular pathways driving tissue damage.

OLP is a common, chronic T-cell-mediated disease with potential for malignant transformation [55]. A recent RNA-seq study by Wang et al. [53] elucidated the transcriptional landscape of OLP, identifying 153 differentially expressed genes (DEGs) enriched in T-cell regulation and Wnt signaling pathways. Key hub genes such as RYK, SLC8A1, WDR7, MAP3K5, and GPBP1 were identified through weighted gene co-expression network analysis (WGCNA), underscoring their potential roles in OLP pathogenesis and offering novel molecular targets for therapeutic intervention. However, the bulk approach could not distinguish whether these transcriptional signatures originated from keratinocytes, infiltrating immune cells, or other stromal components. More recently, Chen et al. [56], by using scRNA-seq, have identified a significantly increased proportion of terminally exhausted CD8^+^ T cells (CD8^+^ T_ex_) in OLP lesions compared to healthy mucosa. The emergence of this exhausted phenotype reinterprets the previously identified “T cell activation” pathways, suggesting they ultimately lead to T-cell dysfunction and providing a mechanistic basis for the disease’s chronicity [57]. A remaining challenge is to determine whether T-cell exhaustion is a primary driver of OLP or a secondary consequence of chronic inflammation.

PV is a classic autoimmune vesiculobullous disease that affects desmogleins (i.e., Dsg1 and Dsg3). Transcriptomic analyses have shown pronounced changes in local tissue milieus as well as systemic immune reactions underlying this disease. Patient-derived autoreactive B cells, particularly after biologic agent treatment such as rituximab exposure, show suppression of pro-inflammatory genes like IL-1β and CD27 [58,59]. This finding suggests an immunomodulatory effect of B-cell-depleting therapies on the cytokine microenvironment, as well as memory B-cell activation patterns. Long non-coding RNAs (lncRNAs) are also increasingly acknowledged in the context of autoimmune pathogenesis. In PV, the lncRNA LINC01588 modulates T cell polarization by acting through the PPAR signaling pathway, directly affecting the critical balance between pro-inflammatory Th17 cells and regulatory Tcells (Tregs) [60]. While these findings highlight a potential regulatory role, it is still debated whether lncRNA dysregulation is a primary driver of T-cell imbalance or a secondary consequence of the ongoing inflammatory process. These regulatory RNAs, in particular, may represent potential biomarkers and therapeutic targets, though their pathophysiological significance and clinical translatability require further validation.

In oral MMPg, disease pathogenesis is primarily driven by autoantibody-mediated targeting of hemidesmosomal components, such as BP180. While transcriptomic data derived from oral lesions are currently scarce, analyses of ocular MMPg transcriptomes offer an informative comparative framework. NanoString transcriptomics was recently employed by Panthagani et al. [61] on biopsies of conjunctival membrane and revealed an upregulation of MMP-9, CXCL10 expression, and of epithelial barrier markers, thereby illuminating ongoing transcriptional remodeling of mucosal disease. These findings highlight ongoing transcriptional remodeling in mucosal disease and support a model in which MMPs contribute not only to antibody-mediated basement membrane destruction but also to stromal and epithelial transcriptional reprogramming, thereby perpetuating chronic inflammation and disrupting tissue homeostasis. However, RNA sequencing or NanoString analysis of oral mucosal biopsy are necessary to verify such signature within oral disease.

Transcriptomic studies have also provided substantial insight into SS, a disorder marked by lymphocytic infiltration of the lacrimal and salivary glands and consequent secretory dysfunction. Gene expression of biopsy samples of labial salivary glands always shows an enhanced type I interferon (IFN) signature with an exaggerated upregulation of MX1, IFIT3, and OAS1. However, the clinical heterogeneity of SS is not fully captured by this signature, as its intensity can vary significantly among patients and does not always correlate directly with specific clinical symptoms or disease progression. Verstappen et al. [62] carried out RNA sequencing of paired parotid and labial gland tissues, reporting an increased enrichment of IFN-α signaling pathways of biopsy-positive primary SS with increased MX1 expression that was related to the systemic blood markers (MxA). This confirms systemic IFN involvement, though its precise triggers and cellular origins (epithelial cells versus hematopoietic cells) are still debated. An earlier NanoString transcriptomic analysis highlighted increased expression of IFI44L, IFI44, OAS1, IFIT3, and MX1 within the epithelial cells of the salivary glands with an indication of an IL-7/IFN amplification loop of the SS pathogenesis [63]. These interferon-stimulated genes not only amplify local glandular inflammation but are also associated with extraglandular symptoms and B-cell hyperactivity, suggesting a potential use for patient stratification as well as disease progression biomarkers.

From a treatment perspective, transcriptomic data has significant potential for precision medicine. However, the current application of transcriptomics is still mainly confined to research, limited by high costs and analytical complexity. In the near future, its clinical impact will be in enriching histopathology, providing prognostic insights and, crucially, guiding the use of targeted biological therapies.

### 3.2. Transcriptomics in Oral Cancer Microenvironment

Tumor microenvironment (TME) of OSCC is a dynamic and complex ecosystem comprising a diverse array of cellular and structural elements, including malignant epithelial cells, stromal cells, immune cell infiltrates, fibroblasts, endothelial cells, and the extracellular matrix [64]. Understanding the molecular interactions that take place within the TME provides essential insight into the tumor’s behavior, its potential for metastasis, and treatment resistance. Transcriptomics, specifically by the technologies of bulk RNA-seq as well as scRNA-seq, has become an effective method for examining cellular components and gene expression profiles that exist within the OSCC microenvironment [65,66].

Bulk transcriptomic analyses have classically assisted in subclassifying OSCC into molecular subtypes that impart discrete biologic behaviors and prognostic outcomes. These analyses have revealed differential gene expression that involves epithelial–mesenchymal transition (EMT), angiogenesis, immune subversion, and extracellular matrix remodeling. Of significant clinical interest is the characterization of partial EMT (p-EMT), a program that allows tumor cells to become invasive while maintaining certain epithelial properties [67]. This p-EMT state is marked by high expression of genes such as VIM, ZEB1, and TWIST1, which are directly correlated with nodal metastasis and adverse clinical endpoints [68].

The development of scRNA-seq has improved the understanding of OSCC tumors’ cellular heterogeneity. This technique allows the analysis of gene expression at the single cell level, thus making it possible to characterize selective subpopulations of cancer cells, cancer-associated fibroblasts (CAFs) [69], tumor-infiltrating lymphocytes (TILs) [70], and myeloid-derived suppressor cells (MDSCs) [71]. Building on single-cell resolution, spatial transcriptomics further enhances our understanding by preserving the spatial context of gene expression within the tissue. Unlike scRNA-seq, spatial transcriptomics maps gene expression to specific locations in the tumor, allowing researchers to visualize cellular interactions and microenvironmental niches. This approach has been particularly valuable in identifying how invasive p-EMT cells, CAFs, and immune populations are organized and communicate within the OSCC microenvironment.

In addition, transcriptomic approaches have defined the contribution of CAFs to the immunosuppressive environment of OSCC. CAFs produce cytokines and chemokines like IL6, CXCL12, and TGF-β that recruit Tregs, suppress the activity of cytotoxic T cells, and drive fibrosis [72]. These interactions not only create an environment of tumor immune evasion but also dictate resistance to immunotherapy. New evidence indicates that the transcriptomic signatures of specific CAF subtypes, namely myofibroblastic and inflammatory, may be used to forecast varying treatments with immune checkpoint inhibitors [73]. Despite this, the field is affected by the significant plasticity and heterogeneity of CAFs, which often complicates the distinction between subtypes and challenges the development of universal CAF-targeted therapies.

Transcriptomic studies have also provided a comprehensive profile of the immunological compartment in the OSCC tumor microenvironment. TILs, including CD8+ T cells, Th17 cells, and exhausted T cells expressing PD-1 and TIM-3, have variable gene expression profiles typical of either immune surveillance or dysfunction [74]. These transcriptional differences have direct clinical implications. For instance, tumors with an “inflamed” gene signature, including high expression of IFNG, GZMB, and CXCL9, are most responsive to immunotherapy while “cold” tumors that have poor immunocyte infiltration are therapeutically recalcitrant [75]. Moreover, transcriptomic profiling has enabled the identification of novel immunoregulatory molecules, including LGALS9, LAG3, and TIGIT, which are currently being explored as potential targets for PD-1/PD-L1 blockade [76,77].

Based on the evidence presented so far, the extreme complexity, dynamism, and heterogeneity of OSCC tumors are evident. This underscores the need to integrate transcriptomic analyses with other omics approaches, advanced computational models, and experimental systems to comprehensively characterize tumor biology, identify novel biomarkers and therapeutic targets, and ultimately guide the development of more personalized treatment strategies. Integration of transcriptomics with spatial transcriptomics as well as multiplex immunohistochemistry are already being used to visualize the interaction as well as spatiotemporal distribution of immune cells within the tumor niche. Then, from a strict clinical practice standpoint comprehensive transcriptomic profiling is not yet a standard diagnostic, prognostic, or predictive tool in the routine management of OSCC. Its clinical impact is yet indirect, fueling the discovery of actionable biomarkers (like PD-L1) and providing the biological rationale for new drug targets.

### 3.3. Summary and Future Directions

Transcriptomics provides a dynamic snapshot of cellular activity, revealing the active molecular processes that underpin oral diseases (Table 2), including disease-specific immune dysregulation, cellular heterogeneity, and therapeutic responses. In autoimmune diseases, transcriptomics has delineated specific immune cell states and signatures, providing a molecular subclassification of diseases. In OSCC, it has uncovered critical programs like partial EMT and defined the complex interactions within the tumor microenvironment that drive invasion and immune evasion. The growing application of single-cell and spatial transcriptomics promises to further define these signatures, paving the way for their use as biomarkers for early diagnosis, patient stratification, and monitoring treatment efficacy.

## 4. Proteomics in Salivary Diagnostics and Tissue Analysis

**Figure 3:**
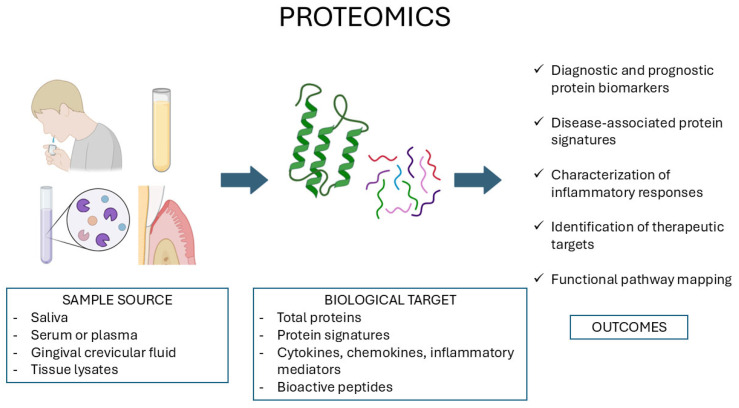
Proteomic analyses of saliva, serum or plasma, GCF and tissue lysates aim to detect proteins, peptides, and inflammatory mediators relevant to oral health. This approach aims to identify diagnostic and prognostic biomarkers, characterize host responses in periodontal and mucosal diseases, and reveal protein-level mechanisms underlying oral pathology.

Proteomics involves large-scale characterization of protein structure, function, and interactions that are the predominant determinants of cellular activity. Although the genome is relatively invariant, the proteome is dynamic and fluctuating in response to disease and external stimuli. Proteomic technologies aim at deciphering such complexity by identifying and quantifying proteins within various biological samples, including cells, biofluids such as saliva, and cell cultures (Figure 3).

Contemporary proteomics relies primarily on mass spectrometry (MS)-based platforms [78]. Common strategies involve the separation of proteins by two-dimensional gel electrophoresis (2D-GE), followed by identification and quantification using liquid chromatography–coupled tandem mass spectrometry (LC–MS/MS). These technologies, however, have limited ability to discriminate post-transcriptional modifications (PTMs), like phosphorylation or glycosylation, which are of essential value for protein functionality and signaling processes. Recent advances in targeted and enrichment-based MS workflow, such as phosphoproteomics, glycoproteomics, and top-down proteomics, have greatly improved the detection and site-specific characterization of PTMs.

Advances of quantitative proteomics have also been enabled by labeling approaches such as isobaric tags for relative and absolute quantification (iTRAQ) and tandem mass tags (TMT), which allow simultaneous measurement of protein expression across multiple samples [79,80]. On the other hand, label-free quantification (LFQ) approaches provide a flexible and cost-effective alternative, relying on spectral counting or mass spectrometry signal intensity–based measurements [81]. More recently, data-independent acquisition (DIA) and sequential window acquisition of all theoretical mass spectra (SWATH-MS) have emerged as accurate and reproducible proteomic profiling strategies [82,83]. These approaches enhance data completeness across cohorts and are particularly valuable for clinical biomarker discovery.

Proteomic analysis now extends beyond the examination of bulk samples. Emerging methodologies, including single-cell proteomics [84] and spatial proteomics [85], are currently under development, facilitating the investigation of protein heterogeneity at both the cellular and tissue levels, allowing protein localization within complex microenvironments.

The translation of proteomic data is finally dependent on bioinformatics pipelines. These pipelines are essential to transform raw spectral data into interpretable protein profiles, and are based on specialized tools like UniProt, PeptideAtlas, and the Human Protein Atlas [86,87,88].

### 4.1. Proteomics of Oral Cancer

Proteomic analyses have enabled the identification of functional dysregulations that arise during carcinogenesis, tumor progression, metastasis, and the development of therapeutic resistance [89]. Proteomics technologies such as 2D-GE, LC-MS/MS, and iTRAQ have been applied to identify and quantify proteins in OSCC tissues, saliva, extracellular vesicles (EVs), and serum samples [90], enabling the detection of differential protein expression between malignant and normal samples, supporting biomarker discovery and molecular pathway profiling.

Among the most significant findings is the overexpression of proteins involved in cellular proliferation, apoptosis resistance, and immunomodulation. A prominent example is Epidermal Growth Factor Receptor (EGFR), which is frequently upregulated in OSCC and serves both as prognostic marker and as an attractive target for treatment [91]. Despite this, the variable response to anti-EGFR therapies highlights that protein abundance alone is an insufficient predictor of therapeutic efficacy, underscoring the need for proteomic strategies that capture activating mutations and post-translational modifications. Other overexpressed heat shock proteins such as Hsp70 and Hsp27 also play their role towards cancer cells’ survival under stress situations [92]. Similarly, aberrant expressions of other proteins such as Annexin A1 [93], Cyclophilin A [94], and α-enolase [95] have been implicated in promoting tumor progression and invasion.

Although several studies have demonstrated the diagnostic and prognostic relevance of cytokeratin (CK) expression patterns in OSCC, current evidence is largely derived from immunohistochemical analyses rather than true proteomic investigations. Specific CK subsets are differentially expressed in OSCC, reflecting distinct degrees of epithelial differentiation and supporting the existence of heterogeneous molecular pathways of tumorigenesis [96]. These findings, however, remain semi-quantitative and spatially limited, and CK profiles are not yet validated diagnostic or prognostic tools in routine clinical practice. To date, comprehensive mass spectrometry-based proteomic profiling focused specifically on CK in OSCC is lacking. Such studies would be essential to elucidate the full spectrum of CK isoforms involved in cytoskeletal remodeling and epithelial plasticity in carcinogenesis, as well as post-translational modifications and interaction networks.

Saliva has emerged as an attractive biofluid for OSCC biomarker discovery due to its non-invasive collection and the high abundance of locally secreted proteins. Proteomic analyses of saliva from OSCC patients have revealed significant alterations in proteins such as Complement Factor H (CFH), Fibrinogen alpha chain (FGA), and Alpha-1-antitrypsin (SERPINA1) [90]. Panels comprising these proteins have demonstrated enhanced sensitivity and specificity for distinguishing OSCC from both healthy controls and individuals with potentially malignant oral disorders [97]. Despite the promising results, salivary proteomic panels for OSCC remain in a pioneering phase. A significant limitation for clinical application is the high inter-individual variability in salivary composition and the influence of common confounders such as oral inflammation, which can lead to false-positive results. EVs derived from OSCC have been found to contain oncogenic proteins and to mediate cell-to-cell communications within the tumor microenvironment [98]. Proteomic characterization of saliva-derived EVs has identified proteins involved in epithelial–mesenchymal transition (EMT), immune modulation, and extracellular matrix remodeling, highlighting their potential as a source of novel, non-invasive biomarkers [99,100]. The current limitation is isolating specific EV subpopulations and deciphering the functional significance of their complex content in tumor progression.

Recent comparative proteomic analyses have also been able to differentiate OSCC metastases in the lung from primary lung squamous cell carcinoma (LUSC) by employing machine learning models trained on typical protein expression profiles [101]. This would represent a critical diagnostic advance allowing the differentiation of primary from metastatic lesions.

Along with biomarker identification, proteomic data have defined multiple dysregulated pathways of OSCC such as PI3K/AKT/mTOR [102], NF-κB signaling pathway [103], oxidative stress response pathway [104], and cytoskeletal remodeling pathway [105]. Besides enhancing the insight of OSCC biology, they also present novel potential therapies, including small-molecule inhibitors, monoclonal antibodies, or combined regimens aimed at overcoming resistance mechanisms.

From a clinical standpoint, direct application of proteomic results in practice has several challenges and, to date, remains unrealized. As noted by Mischak et al. [106], the application of proteomic data in clinical environments is hindered by problems of sample manipulation, analytical results’ reproducibility, and variability across laboratories. Nonetheless, developments of quantitative proteomics and bioinformatics continue to address these limitations [107], while some proteomic discoveries are already used for delivering protein-based biomarkers.

### 4.2. Proteomics in Periodontal Disease

Proteomic studies in periodontal research have primarily focused on gingival crevicular fluid (GCF), saliva, gingival tissues, and more recently EVs. GCF is considered a key diagnostic fluid as it accurately reflects the biochemical environment of the periodontal pocket and contains a broad array of proteins derived from both the host and resident bacteria [108]. MS-based techniques like LC-MS/MS have characterized GCF proteins, including those implicated in immune defense, inflammation, extracellular matrix remodeling, and tissue repair [109]. Among the most notable protein biomarkers in periodontitis are matrix metalloproteinases (MMPs), particularly MMP-8 and MMP-9, which contribute to the degradation of collagen and other extracellular matrix components [110]. Elevated MMP concentrations in GCF are strongly related to continued periodontal tissue destruction making them reliable indicators of disease activity.

In addition to MMPs, other inflammatory mediators like IL-1β, IL-6, TNF-α, and prostaglandin E2 (PGE2) have been consistently found at higher concentrations in patients with periodontitis [111]. Beyond their role in the inflammatory cascade, these cytokines also regulate osteoclast activity and bone resorption, perpetuating advanced periodontal disease. It is important to note, however, that while these mediators are constantly elevated in periodontitis, they represent a common final pathway of inflammation, thus their utility as specific diagnostic biomarkers is limited by their ubiquity in multiple inflammatory conditions.

Proteomic studies have further identified acute-phase proteins, including haptoglobin, alpha-1-antitrypsin, and transferrin, as significant players in periodontal inflammation [112]. These proteins are thought to reflect systemic inflammatory reactions that may link periodontal disease to systemic conditions, such as cardiovascular disease and diabetes, through chronic low-grade inflammation affecting vascular and metabolic pathways. While this supports the “systemic inflammation” hypothesis, establishing direct causal links through proteomic data remains challenging, as these proteins are non-specific responders to a wide range of inflammatory conditions.

Saliva proteomics represents a promising non-invasive approach for diagnosing and monitoring periodontitis. Saliva contains a complex mixture of proteins derived from both salivary glands and gingival crevicular fluid, providing a comprehensive snapshot of oral health. Using SWATH-MS proteomics, a recent study [113] has identified eight novel salivary protein biomarkers, including keratin, type II cytoskeletal 1, protein S100-A8, β-2-microglobulin, neutrophil defensin 1, lysozyme C, ubiquitin-60S ribosomal protein L40, isoform 2 of tropomyosin α-3 chain, and resistin, which demonstrated excellent diagnostic accuracy for periodontitis, with predictive models showing a significant performance increase when adjusted for the patient’s age.

In addition, proteomic characterization of gingival tissues has given an insight into the molecular reorganization of periodontal connective tissue upon exposure to bacterial infections. For instance, proteins involved in oxidative stress, apoptosis, and antigen presentation are upregulated [114], confirming that tissue destruction in periodontitis results not only from direct bacterial effects but also from immunologically mediated mechanisms.

The analysis of salivary proteome has also been proposed for evaluating the effects of periodontal treatment. In a recent prospective study by Silbereisen et al. [115], post-treatment samples showed a reduction in pro-inflammatory mediators and a recovery of proteins involved in repair of the tissues and homeostasis. Despite the small sample size and the absence of a control group, these preliminary findings suggest the potential of the salivary proteome to reflect the dynamic biological processes of wound healing and inflammation resolution following periodontal therapy.

Though the application of proteomics in periodontitis has yielded a rich catalog of potential biomarkers in GCF, saliva, and tissue, the translation of these discoveries into validated, routine diagnostic or prognostic tests has been limited. Challenges include variability in sample collection, reduced protein concentrations in oral fluids, and the requirement for bioinformatic platforms for large-scale data analysis. Overcoming these obstacles is critical for translating proteomic findings into reliable clinical diagnostics and monitoring strategies.

### 4.3. Summary and Future Directions

Proteomics bridges the gap between genetic potential and functional phenotype by directly characterizing the effector molecules in oral diseases (Table 3). The proteome of accessible biofluids like saliva and gingival crevicular fluid holds a rich source of potential biomarkers. In OSCC, mass spectrometry has identified numerous proteins involved in proliferation, invasion, and immune modulation, forming the basis for potential diagnostic panels. In periodontitis, proteomic profiling of inflammatory mediators and tissue-destructive enzymes like MMPs can provide a quantitative measure of disease activity and response to therapy. However, a translational gap exists for most genes, and a direct clinical application of proteomic findings remains largely unrealized. The future challenge for oral proteomics is the clinical validation and standardization of biomarker panels.

## 5. Metabolomics: Metabolic Fingerprinting of Oral Disease

**Figure 4:**
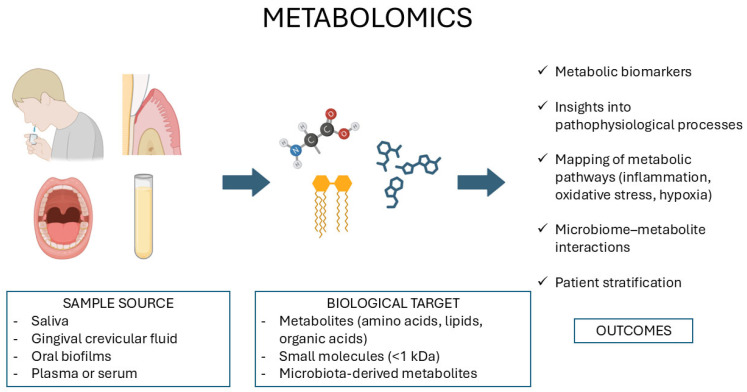
Metabolomic profiling of saliva, serum, GCF, or oral biofilms uncovers metabolic alterations associated with oral inflammation, dysbiosis, and neoplastic transformation. Metabolomics identifies small-molecule biomarkers and metabolic pathways that contribute to the etiopathogenesis and clinical variability of oral diseases.

Metabolomics entails the systematic characterization of small-molecule metabolites (amino acids, organic acids, lipids, and sugars), describing the prevailing biochemical reactions within cells, tissues, or biofluids [116]. These low-molecular-weight entities reflect the end products of gene expression, protein activity, and environmental interactions, placing metabolomics at the center of systems-level approaches for capturing the functional phenotype of a biological system in real time (Figure 4). By bridging the gap between genotype and phenotype, metabolomics supports the development of precision diagnostics and individualized therapeutic strategies [117]. At present, metabolomic studies in dentistry have been conducted mainly on OSCC and periodontal diseases, with minimal investigation into other oral diseases.

Nuclear magnetic resonance (NMR) spectroscopy and MS represent the two most employed analytical platforms in metabolomic analysis, often coupled with chromatographic separation technologies such as gas chromatography (GC) and liquid chromatography LC [118]. NMR provides metabolite quantification with high reproducibility and minimal sample processing requirements [119], whereas MS technologies offer superior sensitivity and extensive metabolite coverage, allowing the identification of hundreds to thousands of metabolites in a single analysis [120]. In addition, new technologies such as capillary electrophoresis–mass spectrometry (CE-MS) and ultra-performance liquid chromatography-mass spectrometry (UPLC-MS) further improve analytical resolution and throughput, advancing metabolomics toward routine clinical and research applications [121].

Common sample types in dental metabolomics include saliva, GCF, dental plaque samples and biopsies of oral tissue, each offering a distinctive view of systemic or localized states of metabolism. Saliva is increasingly regarded as a readily accessible biofluid that can offer insights into both oral and systemic health [122], though its composition is subject to a significant variability depending on the disease status, and its clinical application faces different challenges. Factors like sample collection method (stimulated vs. unstimulated saliva), circadian rhythms, recent food intake, hydration status, oral hygiene, smoking, and local inflammation, in fact, constantly alter the metabolic profile of saliva. Standardized operating procedures for sample collection, processing, and storage are therefore imperative to ensure the accuracy and reproducibility of this method.

Metabolomic data are typically assessed by advanced bioinformatics workflows to discern metabolite profiles, disease-specific biomarkers, and changed pathways of metabolism. Multivariate statistical approaches, such as principal component analysis (PCA) and partial least squares discriminant analysis (PLS-DA), are then used within such analyses to discriminate between disease status and predict clinical outcome.

### 5.1. Metabolomics of OSCC

Unlike genomics and transcriptomics, which provide insights into potential biological functions, metabolomics captures the real-time status of the organism’s functional phenotype. This characteristic makes it particularly valuable for the identification of reliable biomarkers and for the assessment of treatment efficacy.

One of the most characteristic metabolic alterations of OSCC is the “Warburg effect”, characterized by enhanced aerobic glycolysis despite sufficient oxygen availability [123]. Cancer cells preferentially metabolize glucose to lactate, resulting in altered energy production and acidification of the tumor microenvironment. Metabolomic profiling has confirmed high levels of lactate, pyruvate, and glucose-6-phosphate in OSCC tissues and body fluids, confirming this metabolic reprogramming toward glycolysis [124]. These metabolites are found in saliva, plasma, and biopsy specimens, suggesting their potential utility as non-invasive biomarkers for early-stage diagnosis. However, the non-specific nature of several of these metabolites limit their diagnostic specificity, as similar metabolic alterations can occur in other pathological or systemic conditions. Notably, many metabolites can also originate from bacterial fermentation or dysbiosis, making their diagnostic specificity questionable. In this regard, integrating metabolomics with microbiomics and transcriptomics will be essential to attribute metabolic signatures to their biological sources.

Beyond carbohydrate metabolism, the metabolic processing of amino acids was found altered in OSCC [125,126]. Studies employing NMR spectroscopy and MS-based approaches have identified altered levels of glutamine, glutamate, and branched-chain amino acids (BCAAs), such as leucine and valine [127]. Among these, glutamine is of particular interest, being a critical source of carbon and nitrogen for rapidly proliferating tumor cells, supporting nucleotide and amino acid biosynthesis, maintaining redox homeostasis, and modulating mTOR signaling pathways [124].

Beyond the reprogramming of core amino acid metabolism, the dysregulation of specific immunomodulatory pathways has emerged as a metabolic feature of OSCC. Notably, the tryptophan-kynurenine pathway is frequently upregulated in the TME [128]. The rate-limiting enzymes indoleamine 2,3-dioxygenase 1 (IDO1) and tryptophan 2,3-dioxygenase (TDO2) convert tryptophan into kynurenine and its downstream metabolites. This shift has a dual immunosuppressive effect: it depletes local tryptophan, essential for T-cell proliferation and function, while accumulating kynurenine and other metabolites that directly promote the differentiation of regulatory T cells (Tregs) and induce apoptosis of effector T cells [129]. The targeting of this pathway represents an active area of investigation for cancer immunotherapy, highlighting how metabolomic profiling can reveal targetable immune-metabolic nodes within the TME.

Tricarboxylic acid cycle (TCA) and its associated intermediaries have demonstrated significant alterations in OSCC [130]. Reduced synthesis of citrate and succinate with fumarate and malate accumulation indicates a redistribution of mitochondrial metabolism [131]. Such metabolic reprogramming represents not only a consequence of tumor progression but also an adaptive response that meets the increased biosynthetic and energetic demands of cancer cells, thereby supporting their survival under hypoxic and nutrient-deprived conditions.

On this basis, salivary metabolomics has been proposed for the early and non-invasive diagnosis of OSCC [132]. Saliva contains a variety of metabolites that are indicative of both systemic and local physiological changes. Capillary electrophoresis-time-of-flight-mass spectrometry (CE-TOF-MS) and GC-MS have identified specific metabolic signatures, including altered phenylalanine, tryptophan, and their respective downstream catabolites levels [133]. These perturbations are indicative of enhanced protein turnover, immune modulation, and dysregulation of aromatic amino acid metabolism associated with malignant transformation. Despite the promising results, the field is currently in a discovery and validation phase and the translation into a clinically reliable test still necessitates studies with adequate designs, standardized protocols, and large, independent cohorts [134].

In addition to diagnosis, metabolomics may provide predictive insights for treatment response and prognosis. Recent analysis revealed that patients featuring higher pre-treatment concentrations of lactic acid, glutamic acid, and selected TCA intermediates tend to positively respond to induction chemotherapy by docetaxel, cisplatin, and 5-fluorouracil (TPF regimen) [135]. On the other hand, high concentrations of 3-methylhistidine and 5-hydroxylysine in saliva have been linked with adverse overall survival and could be used as prognostic biomarkers [136]. Moreover, novel metabolomic platforms that integrate data from circulating tumor cells and extracellular vesicles aim to detect tumor-derived metabolic fingerprints with enhanced sensitivity [137]. When combined with transcriptomics and proteomics datasets, these integrated approaches offer a comprehensive understanding of OSCC biology and may enable personalized therapeutic strategies.

Clinical translation of metabolomic discoveries remains challenging because of heterogeneity of sample handling, analytical platforms, as well as data interpretation, thus the above-described findings remain in the domain of ongoing research. Moreover, while a specific metabolite profile may distinguish cancer patients from healthy controls, detecting a single, actionable enzyme or pathway for targeted intervention remains a major challenge.

### 5.2. Metabolomics in Periodontal Disease

Although microbial biofilms are recognized as the primary etiological factor of periodontitis, disease progression and severity are strongly affected by the metabolites of the host’s response. Hence, metabolomics has emerged as a valuable method for elucidating the biochemical changes associated with periodontal disease.

By employing state-of-the-art analytical methods such as NMR spectroscopy, GC-MS, and LC-MS, a variety of body fluid metabolic alterations have been identified, including saliva, GCF, and serum. These metabolic signatures have revealed disruptions in key biochemical pathways, particularly those of carbohydrate, amino acid, lipid, and nitrogen metabolism [138].

A frequently reported finding in periodontitis is the increased presence of lactate, pyruvate, and short-chain fatty acids (SCFAs) in both saliva and GCF [139]. These metabolites result from anaerobic bacterial metabolic processes and are associated with localized tissue inflammation and dysbiosis [140]. The increased concentrations of these acidic compounds contribute to tissue degradation by reducing pH levels and promoting proteolytic activity.

Furthermore, metabolomic and related analyses have consistently highlighted increased oxidative stress in periodontitis. Elevated levels of biomarkers such as thiobarbituric acid reactive substances (TBARS), indicative of lipid peroxidation, and 8-hydroxy-2′-deoxyguanosine (8-OHdG), a marker of oxidative DNA damage, are frequently detected in the saliva and gingival crevicular fluid of affected individuals [141]. These molecules provide a direct readout of the oxidative damage of host tissues caused by the sustained inflammatory and bacterial infection, directly linking the metabolic state to the pathogenesis of tissue breakdown [142].

Amino acid metabolism is also altered in periodontal disease. Elevated levels of proline, phenylalanine, and tyrosine have been observed in patients with chronic and severe forms of periodontitis [143], reflecting extensive extracellular matrix degradation and accelerated collagen turnover. Furthermore, perturbations in glutamate and arginine metabolism have been linked to immune modulation and nitric oxide production [144]. Despite these findings, reported levels of amino acids vary considerably between studies, suggesting that patient heterogeneity, sampling techniques, and analytical platforms can influence the accuracy of results. While several metabolites, such as acetate, butyrate, lactate, phenylalanine, and valine, have been consistently identified across multiple studies, their expression patterns remain inconsistent [138]. Some investigations report elevated levels of these metabolites in association with periodontitis, whereas others describe a decrease or no specific trend [145]. This lack of reproducibility is likely due to methodological heterogeneity, including differences in biofluid collection procedures, analytical platforms, and patient populations. Such variability currently limits the reliability of these metabolites as robust biomarkers of disease activity.

Finally, metabolomics could serve for evaluating the biochemical impact of periodontal therapy. Emerging evidence suggests that post-treatment metabolic profiles may display a normalization of previously dysregulated metabolites, characterized, for instance, by reduced lactate levels and increased concentrations of anti-inflammatory compounds. A major translational issue is proving acceptable cost-effectiveness and well-defined indications. In other words, metabolomics must offer a clear clinical advantage over the simple, low-cost, and well-established gold standards of periodontal probing and radiography to justify its application in routine practice.

### 5.3. Summary and Future Directions

Metabolomics describes the ultimate functional outcome of physiological and pathological processes by profiling small-molecule metabolites. Oral diseases produce distinct “metabolic fingerprints” in saliva and other biofluids, offering a real-time, non-invasive window into the disease state (Table 4). OSCC is characterized by a profound reprogramming of energy metabolism (e.g., the Warburg effect) and amino acid pathways, revealing potential therapeutic targets. In periodontitis, metabolomics reflects the biochemical interplay between the host and the dysbiotic microbiome, marked by bacterial fermentation products and host-derived inflammatory metabolites. The high sensitivity of metabolomics makes it particularly promising for monitoring therapeutic efficacy, as successful treatment should shift the metabolic profile back towards a homeostatic state. However, challenges concerning the specificity of individual metabolic biomarkers must be addressed, as many metabolite alterations are shared across different inflammatory and neoplastic conditions.

## 6. Multi-Omics Approaches in Dentistry: From Integration to Precision

The integration of genomics, transcriptomics, proteomics, metabolomics, and microbiomics within a multi-omics framework can provide a comprehensive perspective on the biological mechanisms of diseases.

Microbiomics, i.e., the study of the collective genome of microbial communities, does integrate with other omics disciplines and is particularly relevant in dentistry, where the oral microbiome is a key player in both health and disease [146,147]. Dysbiotic shifts in the oral microbiota are the primary etiological factors in dental caries and periodontitis and are increasingly recognized as modulators of the immune response in oral cancer and other mucosal diseases. Beyond their role in local diseases, dysbiotic oral communities have been implicated in a wide range of systemic conditions, including metabolic disorders, cardiovascular disease, and neuroinflammatory processes [148]. Recent integrative multi-omics analyses have in fact demonstrated that shifts in oral microbial networks can influence host transcriptomic and epigenetic signatures [4]. Factors such as diet, smoking, alcohol consumption, and poor oral hygiene have been shown to significantly reshape the composition and metabolic function of the oral microbiome, consequently creating pro-inflammatory profiles that may contribute to systemic low-grade inflammation. The substantial influence of the oral microbiome on the salivary metabolome presents unique translational challenges for metabolomics, in addition to the persistent issues of high pre-analytical variability and limited compound annotation, thus complicating the interpretation of disease-specific metabolic fingerprints. Metabolomics integration with genomics, transcriptomics, proteomics, and microbiomics is then particularly essential to increase diagnostic specificity and to correctly attribute metabolic alterations to host or microbial processes.

**Figure 5:**
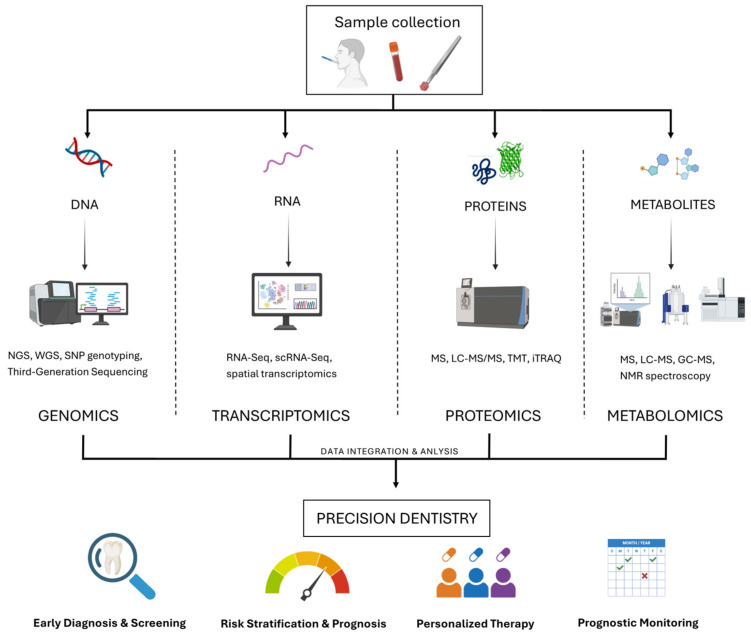
Schematic representation of the multi-omics pipeline in modern dentistry. Biological samples are collected from patients and subjected to high-throughput omics analyses. The resulting large-scale datasets from genomics, transcriptomics, proteomics, metabolomics are integrated using advanced computational and AI-driven approaches. This holistic analysis enables precise clinical applications, including early diagnosis, personalized risk assessment, tailored treatment selection, and ongoing monitoring of oral diseases.

While each omics discipline reveals a specific layer of biological regulation, integrating different datasets is essential for a holistic understanding of diseases [149]. The multi-omics approach involves simultaneous analysis and interpretation of data across different molecular levels and hence captures the dynamics of biological systems more accurately than any single omics level alone [150]. In dentistry, where most conditions are inherently multifactorial, this integrative strategy would represent a useful paradigm shift toward data-driven, precision oral healthcare (Figure 5).

The rationale for adopting a multi-omics strategy lies in the intrinsic interconnectedness of molecular pathways. Genetic variations discovered via genomics can impact gene expression profiles (transcriptomics), which subsequently modify protein levels and functionality (proteomics), ultimately influencing the composition of metabolites (metabolomics) and the resultant clinical phenotype. Conducting an integrated analysis of these distinct layers allows researchers and clinicians to identify crucial molecular determinants, recognize reliable biomarkers, and develop targeted therapeutic strategies.

The value of multi-omics integration has already been demonstrated in oncology. Studies combining transcriptomic and proteomic analyses of tumor tissues and their surrounding stroma have revealed novel molecular subtypes and signaling pathways involved in immune evasion, angiogenesis, and therapeutic resistance [151]. In periodontology, studies integrating host genetic predisposition, gingival gene expression profiles, salivary proteomics, and gingival crevicular fluid metabolomics have provided new insights into host–microbe interactions and inflammatory dysregulation [152].

Methodologically, multi-omics relies on advanced bioinformatics and systems biology platforms capable of managing heterogeneous data types and integrating them into coherent, biologically meaningful models. Network-based analysis, machine learning, as well as dimensionality reduction techniques, are increasingly employed to extract significant molecular patterns [153]. Machine learning and artificial intelligence are increasingly employed to identify patterns, generate predictive analyses of end outcomes, and develop diagnostic algorithms. Given the massive complexity of omics data, the use of these technologies is not simply advantageous but necessary. Moreover, advancements of single-cell and spatial multi-omics are enabling the characterization of transcriptomes as well as proteomes simultaneously at the single-cell level within their microenvironment, offering innovative tools to unravel the heterogeneity and immune landscape of oral lesions [154].

At the clinical practice level, multi-omics drives the development of personalized dentistry, where preventive and treatment strategies are tailored to the molecular characteristics of each individual. For instance, the combination of polygenic risk scores from genomics with proteomics-derived salivary biomarkers and microbiome data could enable the early identification of patients at higher risk of developing periodontitis or caries [155,156,157], allowing focused preventive care. In oral oncology, comprehensive multi-omics tumor profiling could guide the selection of targeted agents, immunomodulatory therapies, or chemotherapeutic regimens according to the unique molecular signature of each patient [158].

Despite the promise, the translation of omics discoveries into routine clinical practice have yet to be realized. Immediate challenges include the standardization of sample collection, processing, and analytical protocols across laboratories to ensure reproducibility. The cost-effectiveness of implementing wide-scale omics screening must also be carefully evaluated for healthcare systems sustainability. Furthermore, the interpretability of complex multi-omics data for clinicians, alongside ethical considerations such as genetic counseling and data privacy, will require the development of clear guidelines.

## 7. Conclusions

Omics sciences are progressively transforming the landscape of oral research and clinical practice. By decoding the molecular basis of diseases, these novel approaches are showing their potential to contribute to more accurate prognosis, individually tailored treatments, and increasingly predictable clinical outcomes, ultimately driving dentistry to a data-driven, preventive, and patient-centered discipline.

Despite their significant potential, several issues limit the full translation of omics sciences into routine clinical practice. The high cost of omics technologies remains a major barrier, particularly for large-scale or longitudinal studies. Considerable variability also exists across analytical platforms, sample preparation protocols, and laboratory workflows, reducing the reproducibility of these techniques. Consequently, most omics-derived biomarkers remain at the discovery or exploratory phase, lacking analytical validation, clinical qualification, and standardization. Finally, ethical and privacy concerns related to genomic data as well as uncertainties in cost-effectiveness further limit the immediate adoption of omics technologies in dentistry.

Future research must focus on longitudinal studies, large-scale integration of multi-omics data, and the development of interoperable and shareable platforms to support clinical translation. We are at the dawn of a new era in medicine and dentistry, in which precision, prediction, and personalization will redefine not only how diseases are treated but how health itself is understood and maintained.

## Figures and Tables

**Table 1:** Key genomic and epigenomic biomarkers associated with OSCC, dental caries and periodontitis.

Oral Disease	Representative Genomic Markers	Type of Alteration	Functional/Clinical Significance	Type of Evidence/Clinical Relevance	References
OSCC	TP53, CDKN2A (p16INK4a), NOTCH1, FAT1, CASP8, HRAS, PTPRT, ARID2, TERT, PIK3CA, PTEN, CD274 (PD-L1), JAK1/2	Somatic mutations, epigenetic alteration, amplifications/deletions, promoter mutations, Pathway-level dysregulation	Loss of tumor suppression, activation of oncogenic pathways, genomic instability, increased proliferation, invasion, metastasis, immune evasion, poor prognosis, therapeutic targeting, prognostic biomarker potential	p16: established clinical biomarker (for HPV). Other markers: discovery-phase omics studies (genomic profiling)	[6,7,8,9,10,11,12,16,17,18]
OSCC (HPV+)	HPV16 E6/E7, CDKN2A/p16	Viral oncogenic integration	Inactivation of p53 and pRB, p16 overexpression, genomic stability, favorable prognosis, predictive marker for HPV-driven subtype	Established clinical biomarker (routine diagnostic test)	[13,14,15]
Dental caries	WNT10A, DEFB1, AQP5, TAS1R2, TAS1R3, AMELX, MMP20	Genetic variants, SNPs, mutations, epigenetic modifications	Impaired enamel formation, defective mineralization, altered salivary composition, dysregulated immune defense, modified taste perception, increased sugar intake, higher caries susceptibility, potential for personalized prevention	Discovery-phase omics studies; preliminary clinical studies	[21,22,3,23,24,25,26,27,28,29,30,31,32,33]
Periodontitis	SIGLEC5, IL1α/β, IL6, TNF-α, TLR4, SIGLEC5, MMP2/8/9, VDR, GLP1R	Genetic polymorphisms, SNPs, functional variants, epigenetic modifications	Exaggerated inflammatory response, impaired immune regulation, increased tissue destruction, enhanced bone resorption, altered host–microbe interactions, higher disease susceptibility, link with metabolic disorders, potential for personalized risk assessment	Discovery-phase omics studies	[34,35,36,37,38,39,40]

**Table 2:** Representative transcriptomic signatures and regulatory RNAs in oral pathology.

Oral Disease	Transcriptomic Biomarkers	Molecular Alteration	Functional/Diagnostic Relevance	Type of Evidence/Clinical Relevance	References
Autoimmune diseases (OLP, PV, MMP, SS)	IRF8, TYROBP, FCER1G, PHGDH, LINC01588, CXCL10, IFI44L, IFIT3, MX1	Upregulated immune and IFN-response genes	Pathogenic Immune Dysregulation & T-cell Exhaustion, Therapy Response & Immune Regulation	Discovery-phase omics studies	[55,53,56,57,58,59,60,61,62,63]
OSCC	VIM, ZEB1, TWIST1, CXCL12, IL6, TGF-β, IFNG, PD-1, LAG3, TIGIT, miR-21, miR-125a, miR-31	Up-regulation and differential gene expression, miRNA overexpression	EMT, CAF activation, immune evasion, therapy response, potential non-invasive early diagnostic biomarkers	Discovery-phase omics studies; no clinical validation	[65,66,67,68,69,70,71,72,73,74,75,76,77]

**Table 3:** Principal proteomic biomarkers identified in OSCC and periodontitis.

Oral Disease	Proteomic Biomarkers	Molecular Alteration	Functional/Clinical Significance	Type of Evidence/Clinical Relevance	References
OSCC	EGFR, Hsp70, Hsp27, Annexin A1, α-enolase, Cyclophilin A, CKs, CFH, FGA, SERPINA1	Overexpression in saliva, tissue, EVs	Cell proliferation, stress response, diagnostic panel candidates	Discovery-phase omics studies	[91,92,93,94,95,96,90,97,98,99,100,101,102,103,104,105]
Periodontitis	MMP-8, MMP-9, IL-1β, IL-6, TNF-α, PGE2, haptoglobin, S100A8, lysozyme C, resistin	Overexpression in GCF and saliva	Inflammation, bone resorption, response to therapy	Discovery-phase omics studies; preliminary clinical studies for evaluating treatment outcomes	[110,111,112,113,114,115]

**Table 4:** Salivary and tissue metabolomic biomarkers in OSCC and periodontitis.

Oral Disease	Metabolomic Biomarkers	Metabolic Alteration/Pathway	Diagnostic/Functional Interpretation	Type of Evidence/Clinical Relevance	References
OSCC	Lactate, pyruvate, glucose-6-phosphate, glutamine, glutamate, valine, leucine, kynurenine, succinate, citrate, fumarate, malate	Warburg effect, amino acid metabolism, TCA cycle	Diagnostic and prognostic biomarkers, therapeutic targets	Discovery-phase omics studies; no clinical validation	[123,124,125,126,127,128,129,130,131,132,133,134,135,136,137]
Periodontitis	Lactate, SCFAs (acetate, butyrate), proline, phenylalanine, tyrosine, glutamate, arginine, TBARS, 8-OHdG	Dysbiosis, collagen degradation, oxidative stress	Reflect inflammatory status and microbial activity	Established markers of oxidative stress from preclinical/in vitro evidence; no clinical validation	[138,139,140,141,142,143,144,145]

## Data Availability

No new data were created or analyzed in this study. Data sharing is not applicable to this article.

## Abbreviations

The following abbreviations are used in this manuscript:

2D-GE	Two-dimensional gel electrophoresis
CAFs	Cancer-associated fibroblasts
CE-MS	Capillary electrophoresis–mass spectrometry
DIA	Data-independent acquisition
DMFS	Decayed, missing, and filled surfaces
EMT	Epithelial–mesenchymal transition
EVs	Extracellular vesicles
GC-MS	Gas chromatography-mass spectrometry
GCF	Gingival crevicular fluid
GWAS	Genome-wide association study(s)
iTRAQ	Isobaric tags for relative and absolute quantitation
LC-MS/MS	Liquid chromatography coupled to tandem mass spectrometry
LFQ	Label-free quantification
lncRNA	Long non-coding RNA
MDSCs	Myeloid-derived suppressor cells
miRNA	MicroRNA
MMPg	Mucous membrane pemphigoid
mRNA	Messenger RNA
MS	Mass spectrometry
NGS	Next-generation sequencing
NMR	Nuclear magnetic resonance
OSCC	Oral squamous cell carcinoma
p-EMT	Partial epithelial–mesenchymal transition
PCA	Principal component analysis
PLS-DA	Partial least squares discriminant analysis
PRS	Polygenic risk score
PV	Pemphigus vulgaris
RNA-seq	RNA sequencing
SCFAs	Short-chain fatty acids
scRNA-seq	Single-cell RNA sequencing
SS	Sjögren’s syndrome
SWATH-MS	Sequential window acquisition of all theoretical mass spectra
TCA	Tricarboxylic acid cycle
TILs	Tumor-infiltrating lymphocytes
TME	Tumor microenvironment
TMT	Tandem mass tags
UPLC-MS	Ultra-performance liquid chromatography-mass spectrometry
WES	Whole-exome sequencing
